# Deep-level defects induced by implantations of Si and Mg ions into undoped epitaxial GaN

**DOI:** 10.1038/s41598-024-65142-w

**Published:** 2024-06-20

**Authors:** Paweł Kamiński, Andrzej Turos, Roman Kozłowski, Kamila Stefańska-Skrobas, Jarosław Żelazko, Ewa Grzanka

**Affiliations:** 1 Łukasiewicz Research Network – Institute of Microelectronics and Photonics, Aleja Lotników 32/46, 02-668 Warsaw, Poland; 2 National Centre for Nuclear Research, ul. Andrzeja Sołtana 7, 05-400 Otwock, Poland; 3 Institute of High Pressure Physics of the Polish Academy of Sciences, ul. Sokołowska 29/37, 01-142 Warsaw, Poland

**Keywords:** Materials science, Nanoscience and technology, Materials for devices

## Abstract

The properties and concentrations of deep-level defects induced by implantations of Si and Mg ions into unintentionally doped (UID) epitaxial GaN have been revealed by using the Laplace-transform photoinduced transient spectroscopy (LPITS) and molecular dynamics (MD) calculations. The material lattice damage, produced by the Si ions implanted at room temperature in the single process at the energies of 200 and 340 keV, is compared with that produced by the Mg ions implanted in the similar process at the energies of 150, 210, and 270 keV. The LPITS results indicate that the same deep traps with the activation energies of 396, 512, 531, 587, 635, and 736 meV are present in the tail regions of the semi-insulating Si- and Mg-implanted films. It is argued that the predominant implantation-induced point defects in the tail region of the Si-implanted films are nitrogen vacancies, whose concentration is 7.7 × 10^17^ cm^−3^. In the Mg-implanted films, the predominant implantation-induced point defects are gallium interstitials, whose concentration is 1.2 × 1 0^18^ cm^−3^.

## Introduction

Epitaxial GaN grown by metal–organic chemical vapor deposition (MOCVD) has recently become an important material for manufacturing a wide range of photonic and high-power electronic devices also operating at high frequencies and high temperatures. These devices are necessary for the development of automotive and aerospace electronics as well as for the modernization of the power generation industry. Nowadays, implantations of Si and Mg ions are key technological operations employed in modern GaN-based device technology allowing for selectively introducing the dopants in order to obtain the desired electrical properties in the near-surface regions of the device^1–3^.

According to molecular dynamics (MD) simulations of the implantation-induced GaN lattice damage, high densities of vacancies and interstitials, resulting from the Frenkel pairs formation, are expected to be present in the films implanted at room temperature with Si and Mg ions at an energy at least of 100 keV and a dose of the order of 10^14^ cm^−2^ used in devices^2–4^. However, the nature of these defects, as well as their properties and concentrations, have been poorly confirmed experimentally.

In the present investigation, the state-of-the-art Laplace-transform photoinduced transient spectroscopy (LPITS), which has been previously used to analyze defect centers in semi-insulating (SI) SiC and proton-irradiated high-resistivity silicon^5,6^, is employed to obtain information concerning the nature, energy levels and the concentrations of point defects created in unintentionally doped (UID) epitaxial GaN by implantations of Si and Mg ions. The present study is the first systematic attempt to identify deep traps associated with implantation-induced native defects present in the unannealed Si- and Mg-implanted films with SI properties. The identification is made by comparing the activation energies of experimentally detected deep traps with the known transition levels of native defects in GaN calculated using the Heyd, Scusseria, and Ernzherof (HSE) range-separated hybrid functional and the projector-augmented wave (PAW) formalism, implemented in the VASP code^7^. The LPITS studies are combined with the MD simulations of the GaN lattice damage induced by the Si an Mg implantations. The results indicate that in the tail regions of the as-implanted semi-insulating epitaxial GaN layers the same deep-level defects are formed. However, the defect concentrations are different, and the mechanism accounting for this phenomenon is discussed.

## Experimental results and discussion

### Dopants concentrations profiles

**Figure 1:**
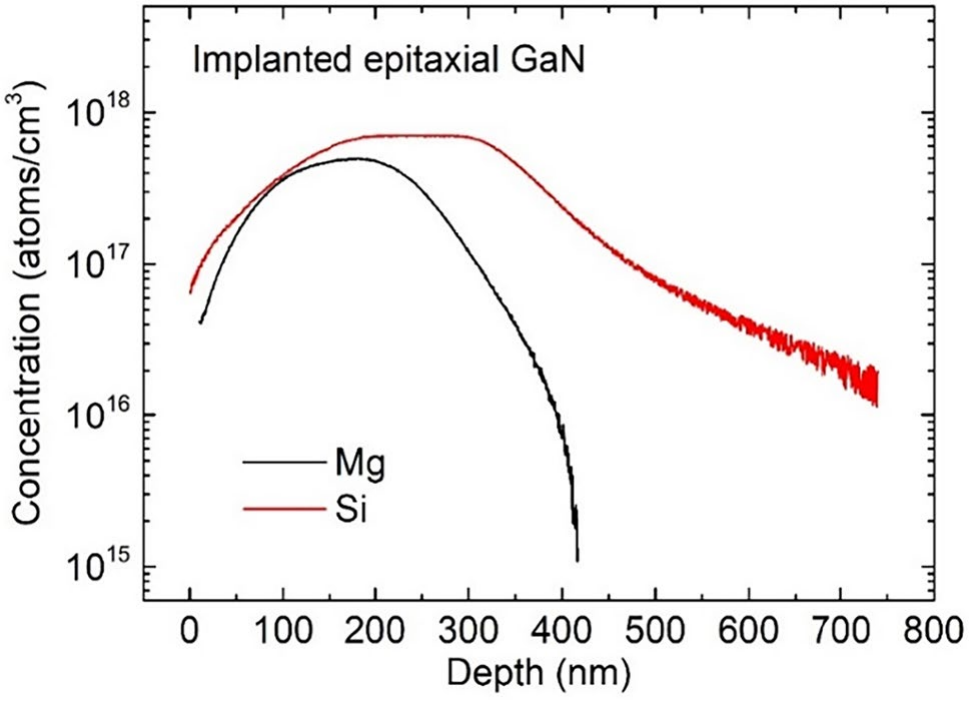
SIMS profiles of Si and Mg concentrations in as-implanted UID GaN epitaxial films.

The SIMS profiles of the Si and Mg concentrations versus depth in the as-implanted UID GaN epilayers are illustrated in Fig. 1.

It is easily seen that the Si concentration distribution is substantially different from that of the Mg concentration. The Si concentration is clearly higher in the whole range of depths, and in the regions located deeper than 300 nm, it exceeds the Mg concentration even by more than one order of magnitude. Within the range of 150–350 nm the former is nearly constant, being ~ 7 × 10^17^ cm^−3^, and this result is in good agreement with the predictions based on Stopping and Range of Ions in Matter (SRIM) simulations. The latter is nearly constant in the narrower range of depths, between 150 and 250 nm, reaching the value of ~ 4 × 10^17^ cm^−3^, which is approximately two times lower than that predicted by SRIM simulations. It is worth adding that the SIMS profile for the concentration of implanted Si ions gives experimental evidence that Si atoms are the residual shallow donors in UID epitaxial GaN films. The influence of the background Si concentration in the film begins to be seen at a depth of 450 nm where the Si concentration is ~ 1 × 10^17^ cm^−3^. The changes in the Si concentration observed in the tail region between 450 and 750 nm suggest that this concentration is related to both the implanted Si atoms and the residual Si atoms in the epitaxial films. At the distance of ~ 500 nm from the film surface the latter seems to be predominant. The Si_Ga_ is the predominant residual shallow donor in epitaxial GaN grown by MOCVD and its background concentration can be in the range of 1 × 10^16^–1 × 10^17^ cm^−3^ ^2,8^. Apart from residual shallow donors, residual shallow acceptors are also present in the material^8,9^. The fact the UID GaN epilayers are usually *n*- type with an electron concentration ranging from 2 × 10^15^ to 5 × 10^16^ cm^−3^ indicates that the residual concentration of shallow donors (*N*_SD_) is slightly higher than the residual concentration of shallow acceptors (*N*_SA_)^8^.

### Properties and concentrations of implantation-induced deep-level defects

**Figure 2:**
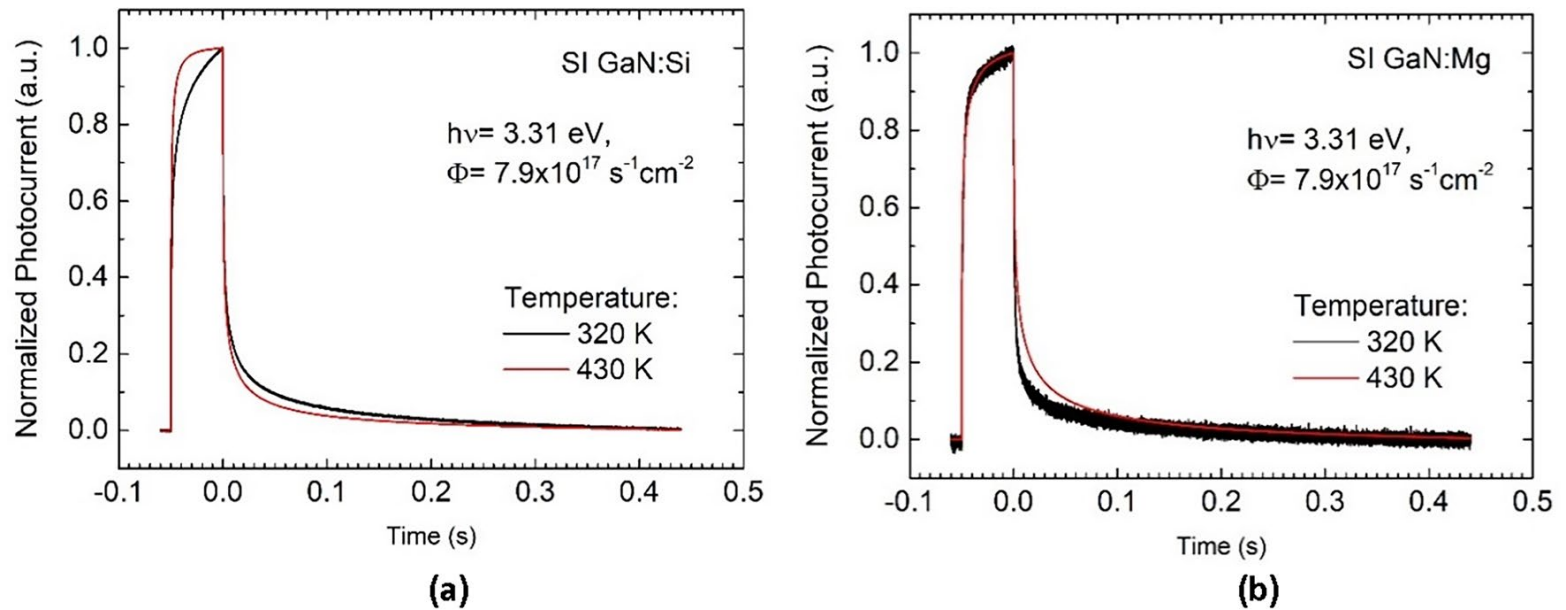
Comparison of normalized photocurrent transients generated at temperatures of 320 and 430 K by the optical excitation in a sample of Si-implanted UID epitaxial GaN (**a**) and in a sample of the Mg-implanted material (**b**).

To determine the characteristics and concentrations of charge carriers traps using the CONTIN procedure^5,6^, a set of the photocurrent transients generated in the Si- and Mg- implanted samples at temperatures 300–520 K was digitally recorded and analyzed. Figure 2 exemplifies the transients measured at temperatures 320 and 430 K for a Si-implanted sample (Fig. 2a) and for a Mg-implanted one (Fig. 2b).

The amplitudes of the photocurrent transients shown in Fig. 2 are normalized with respect of the height of the photocurrent pulse when the sample illumination is switched off. In this way the lower parts of the photocurrent decays related to the thermal emission of excess charge carriers from deep-level defects are exposed. It is easy to see that in the case of the Si-implanted sample, the amplitude of the photocurrent decay observed 320 K is higher than that of observed at 430 K. This fact suggests that in this sample, the concentrations of defects contributing to the thermal emission of charge carriers at the lower temperature are greater than those giving rise to the thermal emission at the higher temperature. In other words, the activations energies of the former are lower than that of the latter. In contrast to the decays shown in Fig. 2a, the amplitude of the photocurrent decay observed in Fig. 2b at 320 K is lower than that observed at 430 K. This fact suggests that in the Mg-implanted sample, the concentrations of defects contributing to the thermal emission of charge carriers at the lower temperature are smaller than those giving rise to the thermal emission at the higher temperature.

**Figure 3:**
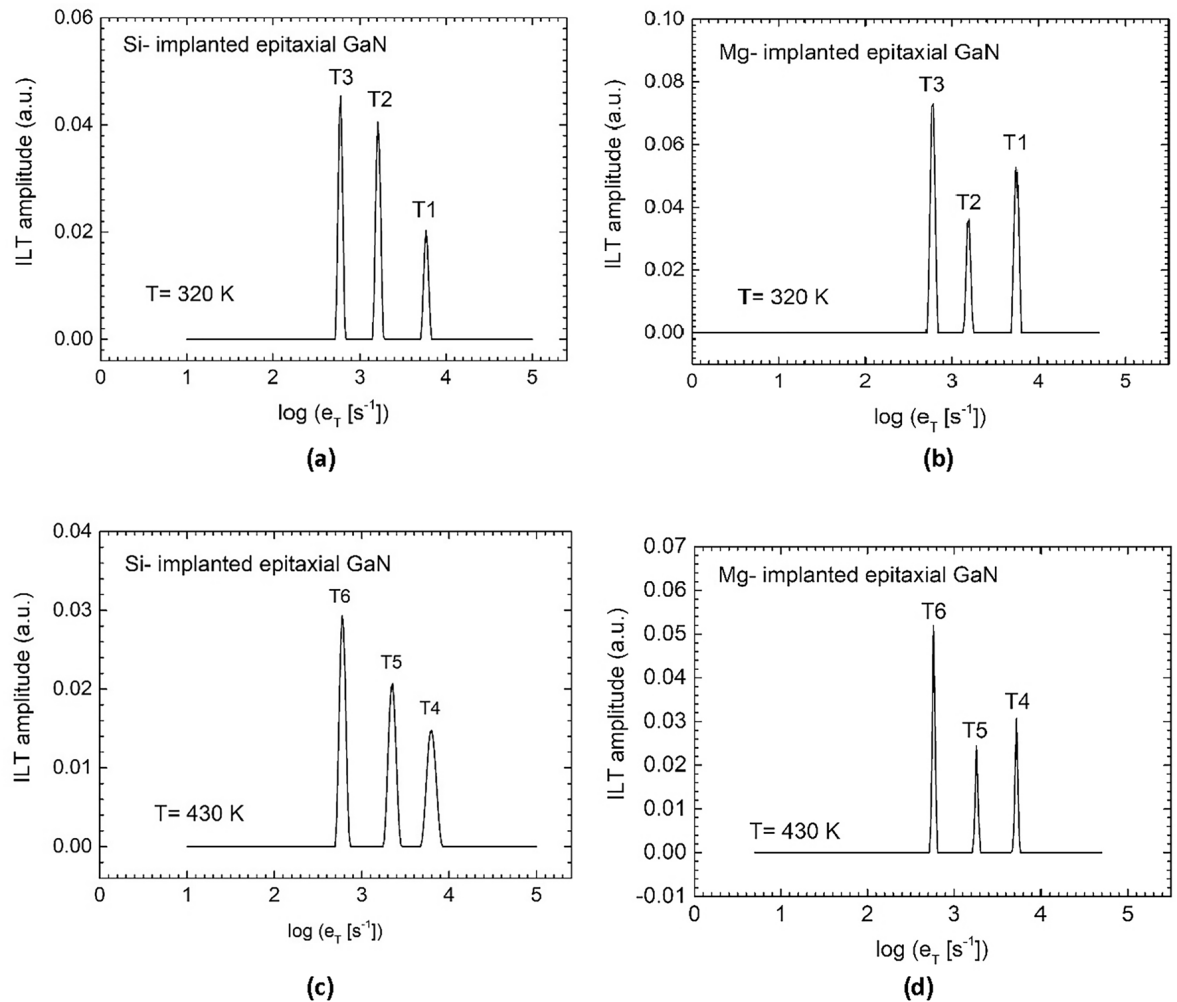
One-dimensional Laplace spectra, derived from the photocurrent relaxation waveforms measured at 320 K for the Si-implanted (**a**) and Mg-implanted UID epitaxial GaN (**b**), indicating the presence of deep traps, labelled as T1, T2, and T3. One-dimensional Laplace spectra, derived from the photocurrent relaxation waveform recorded at 430 K for the Si-implanted (**c**) and Mg-implanted material (**d**), indicating the presence of deep traps, labelled as T4, T5, and T6, with higher activation energies.

Figure 3a,b show the one-dimensional Laplace spectra for the Si- and Mg-implanted samples, respectively, obtained by using the CONTIN numerical procedure to extracting the exponential components in the photocurrent relaxation waveforms representing parts of the transients measured at the lower temperature. Figure 3c,d demonstrate the comparison of the Laplace spectra for these samples, derived from the measurements at the higher temperature. The Laplace spectra shown in Fig. 3a,b consist of three sharp peaks, indicating that in the photocurrent relaxation waveforms recorded at 320 K for the both kinds of samples there are three exponential components whose time constants reciprocals are indicated by the peak positions on the x-axis. These components are associated with the thermal emission of charge carriers from deep traps labelled as T1, T2, and T3, for which the thermal emission rate values at 320 K are 5011.87, 1584.90, and 562.34 s^−1^, respectively. The Laplace spectra shown in Fig. 3c,d also contain three sharp peaks giving evidence that in the photocurrent relaxation waveforms recorded at 430 K for the Si- and Mg-implanted samples there are three exponential components, whose time constants reciprocals are indicated by the peak positions on the x-axis.

 These components are associated with the thermal emission of charge carriers from deep traps labelled as T4, T5, and T6, for which the thermal emission rate values at 430 K are 5623.41, 1995.26, and 602.56 s^−1^, respectively. The fact that the T4, T5, and T6 traps are observed at the considerably higher temperature indicates that the activation energies for the charge carriers emission are higher compared to those for the traps T1, T2, and T3. Thus, it can be assumed that in both the Si- and Mg-implanted UID epitaxial films, the LPITS measurements enabled the six deep traps with increasing values of the activation energy, namely T1, T2, T3, T4, T5, and T6, to be detected. The traps properties were derived from the two-dimensional analysis (in the time and temperature domains) of the photocurrent relaxation waveforms measured at temperatures 300–520 K.

The electronic properties of native point defects identified in the SI films made in UID GaN epilayers by the implantations of Si and Mg ions are summarized in in Table 1. It is worth emphasizing that the atomic configurations of the implantation-induced native point defects given in this table are proposed on the grounds of the experimental results, obtained by LPITS measurements, combined with the recently available results obtained by complex ab initio calculations using hybrid density functionals^7,10^.

**Table 1:** Tentative identification of the deep traps resolved by LPITS in SI samples of Si- and Mg-implanted films in UID epitaxial GaN through comparing the traps activation energies with the energies for the charge state change of native defects calculated by using HSE hybrid functional^7^.

Trap label	Activation energy [meV]	Energy for the charge state change [meV]^7^	Trap identification	Charge state change	Capture cross-section for electrons (*σ*_n_) or holes (*σ*_p_) [cm^2^]	Defect type
T1	396 ± 10	352	Electron trap related to split interstitial N_*i*_–N_*i*_ (−/0)	(-/0) due to electron emission	*σ*_n_ = 1.02 × 10^−16^	Acceptor
T2	512 ± 8	470	Hole trap related to nitrogen vacancy *V*_N_ (2 + / +)	(2 + / +) due to hole emission	*σ*_p_ = 1.25 × 1 0^−16^	Donor
T3	531 ± 8	510	Hole trap related to split interstitial N_*i*_–N_*i*_ (2 + / +)	(2 + / +) due to hole emission	*σ*_p_ = 6.01 × 10^−17^	Donor
T4	587 ± 10	526	Electron trap related to gallium vacancy *V*_Ga_ (3−/2−)	(3−/2−) due to electron emission	*σ*_n_ = 3.24 × 10^−16^	Acceptor
T5	635 ± 10	610	Hole trap related to nitrogen vacancy *V*_N_ (3 + /2 +)	(3 + /2 +) due to hole emission	*σ*_p_ = 4.43 × 10^−17^	Donor
T6	736 ± 8	696	Electron trap related to gallium interstitial Ga_*i*_ (+ /2 +)	(+ /2 +) due to electron emission	*σ*_n_ = 2.00 × 10^−15^	Donor

Furthermore, these atomic configurations perfectly match the predicted by MD simulations microscopic structure of native defects arising due to cascade displacements of gallium and nitrogen atoms under ion implantation^4,11^. The results listed in Table 1 represent the first demonstration of the theoretically predicted electronic properties of native defects, induced by displacements of Ga and N atoms in the GaN lattice under the implantations of Si and Mg ions, in comparison with the electronic properties of deep traps, experimentally determined from the observations and analysis of the relaxation waveforms produced by the thermal emission of electron or holes from the defects present in the as-implanted samples. Because of the small covalent atomic radius (74 pm)^12^, the N_*i*_ are mobile at room temperature and at a sufficiently high concentration, dependent on the ion energy and dose, react with other N_*i*_ atoms to give the two-nitrogen-atom split interstitials^7,10^. According to the presented identification, the N_*i*_–N_*i*_ pairs in the as-implanted samples are in two charge states. They are deep neutral acceptors, which under the excitation of electron–hole pairs capture an electron from the conduction band, as well as they can be singly positively ionized deep donors, which under the samples illumination capture second hole from the valence band. The *V*_N_ are deep donors in two charge states—as singly (trap T2) or doubly (trap T5) positively ionized defects. In the experiment they act as the hole traps, capturing a second or third hole, respectively. It is worth noting that the cross-section for the second hole capture is nearly three times larger than for the third one. The components of Frenkel pairs formed in the Ga sublattice are represented by the *V*_Ga_, being the doubly negatively ionized acceptors and acting in the experiment as the T4 electron trap, as well as by the Ga_*i*_, being the doubly positively ionized donors, observed in the experiment as the T6 electron trap.

It is worth noting that the identification of traps given above allows to account for the various mechanisms of charge compensation in semi-insulating layers produced by the implantations of Si and Mg ions. In the Si-implanted samples of high resistivity, the Fermi level location extrapolated to the temperature of 0 K, found from the temperature dependence of dark current (TDCC) measurements, is at *E*_c_—480 meV. This location is close to the level at *E*_c_—526 meV corresponding to the *V*_Ga_ (3−/2−), which indicates that the gallium vacancies in the (2−) charge state are the main acceptor defects compensating the positively ionized shallow donors related to Si_Ga_. These shallow donors can be formed during the Si-implantation by the interaction of Si interstitials with gallium vacancies. In the semi-insulating Mg-implanted samples, the Fermi level location, extrapolated to the temperature of 0 K, found from the TDCC measurements, is at *E*_v_—745 meV. In this case, the Fermi level position is close to the deep donor level of Ga_*i*_ (+ /2 +) at *E*_c_—696 meV. In view of the results given in Table 1, this fact indicates that in contrast to the Si-implanted material, the charge compensation in the Mg-implanted one is mainly due to the formation of the deep donors Ga_*i*_ (2 +) and the deep acceptors *V*_Ga_ (2−).

### Molecular dynamics simulations results – comparison with experimentally determined deep-level defects properties and concentrations

**Figure 4:**
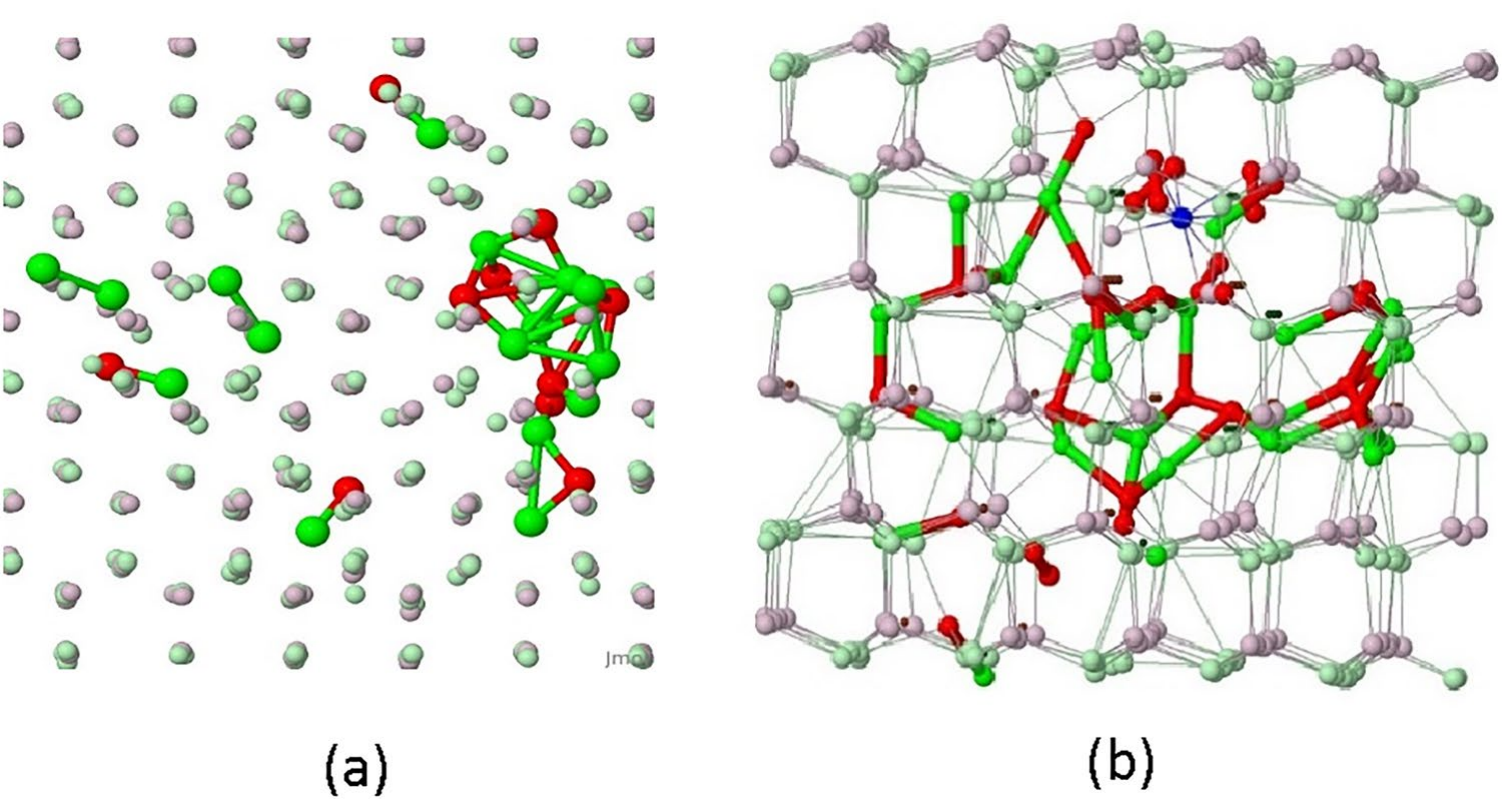
Images of GaN lattice damaged by the collision cascades of Ga and N atoms displaced from the substitutional positions under implantation of Si or Mg atoms. (**a**) Visualization of the isolated defects separated from a defects cluster. (**b**) Visualization of defects inside the cluster. The red and green balls represent the displaced Ga and N atoms, respectively. The pinkish and greenish balls represent, respectively, the Ga and N atoms in the lattice sites. The small dark balls visible inside the cluster represent vacancies.

The images obtained by molecular dynamics simulations illustrating two kinds of damage of GaN crystal lattice induced by the implantation of Si or Mg ions at room temperature are shown in Fig. 4.

The presented-above images give a deeper insight into the defect structure produced in the GaN crystal lattice by the cascade collisions with PKA energies up to 400 keV occurring under Si or Mg implantation. The image in Fig. 4a indicates, that the material structure remains monocrystalline with the presence of isolated point defects as well as point defects clusters. It is worth emphasizing that the isolated defects seen in Fig. 4a are well separated from the cluster. These defects are the N_*i*_–N_*i*_ and Ga_*i*_–N_*i*_ dumbbells formed due to interactions of N_*i*_ and Ga_*i*_ atoms. The gallium and nitrogen vacancies (*V*_Ga_ and* V*_N_) and interstitials (Ga_*i*_ and N_*i*_) are found to be predominant defects in the damaged GaN lattice. In other words the total number of vacancies produced in the Ga and N sublattice is the highest compared to the number of other kinds of defects and it is convenient to express the other defects contribution to the lattice damage by the ratio of the number of particular defects to the total number of vacancies. For the N_*i*_–N_*i*_ and Ga_*i*_–N_*i*_ dumbbells the values of this ratio were found to be 1.2% and 6.3%, respectively. For comparison, the numbers of the single interstitials, Ga_*i*_ and N_*i*_, normalized with respect to the total number of vacancies were found to be 57% and 43%, respectively. It is worth noting that the *V*_Ga_ and* V*_N_ contributions to the total number of vacancies were calculated to be 59% and 41%, respectively.

**Figure 5:**
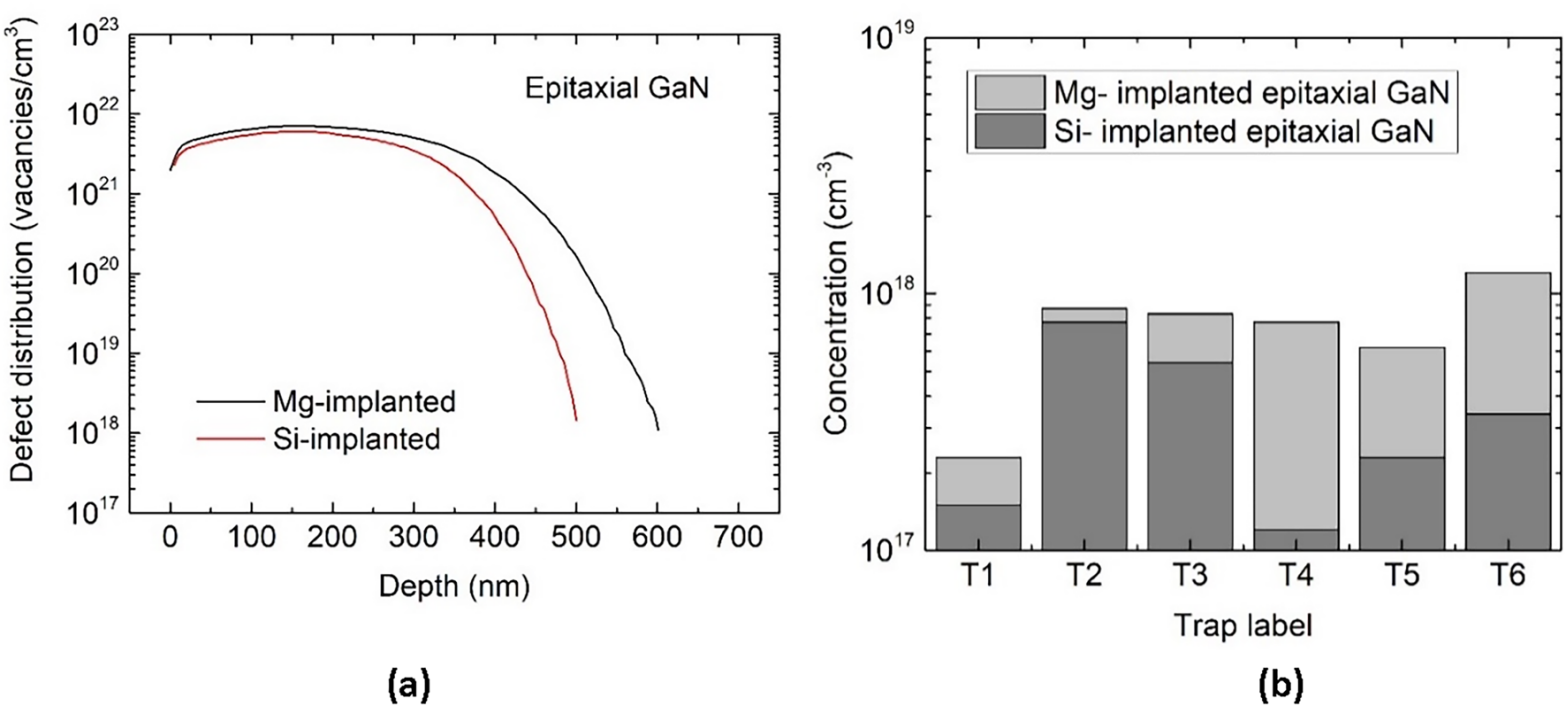
(**a**) Comparison of depth profiles of the sum of *V*_Ga_ and* V*_N_ concentrations determined by means of the MD simulations in Si- and Mg-implanted films made in UID epitaxial GaN. (**b**) Comparison of deep traps concentrations determined by LPITS measurements in the Si- and Mg-implanted SI films made in UID epitaxial GaN.

Figure 5a illustrates the simulated distributions of the total vacancy concentration ([*V*] = [*V*_Ga_] + [*V*_N_]) within the films implanted with Si and Mg ions made in UID epitaxial GaN and Fig. 5b visualizes the values of concentrations of all deep traps detected in the implanted films. The [*V*] distributions shown in Fig. 5a reflect different physical properties of Si and Mg ions implanted at the same energy of 400 keV into the epitaxial GaN films. The main feature of these results is that at the same depth within the implanted layers, the [*V*] values in the Si-implanted layer are clearly smaller than those in the Mg-implanted one. Particularly, this phenomenon begins to be seen at the depth of 200 nm when the [*V*] value in the former represents 71% that in the latter. At the depths of 300 and 400 nm, the total vacancy concentrations in the Si-implanted layer significantly decrease, being 60% and 20% those in the Mg-implanted one, respectively. Interesting facts helping to understand this phenomenon can be derived from the SIMS results (Fig. 1) showing the penetration depths of Si and Mg ions in the implanted samples. These results indicate that at the same depths, the Si concentration ([Si]) in the Si-implanted layer is higher than the Mg concentration ([Mg]) in the layer implanted with Mg ions under similar conditions. At the depths of 200, 300, and 400 nm, the [Si]/[Mg] ratio values are 1.4, 7.0, and 30, respectively. Thus, it can be concluded that although the penetration depth of Si ions in epitaxial GaN is clearly greater than that of the Mg ions, the total vacancy concentration produced by the former is substantially lower than that produced by the latter. Using the [Si] and [Mg] values given in Fig. 1 and the [*V*] values shown in Fig. 5a at the particular depths in the Si- and Mg-implanted epitaxial GaN it is easy to estimate the numbers of *V*_Ga_ and *V*_N_, remained in the material after the formation of the substitutional Ga and N atoms displacements cascades, induced by the collisions of single Si and Mg ions reaching the depth of 200, 300, and 400 nm and the defects recombination. In this way, the numbers of all vacancies remained at these depths after the cascades triggered by a Si ion are 7.14 × 10^3^, 4.29 × 10^3^, and 1.33 × 10^3^, respectively. The numbers of all vacancies remained at these depths after the cascades triggered by a Mg ion are much higher and equal to 1.4 × 10^4^, 5.0 × 10^4^, and 2.0 × 10^5^, respectively. It is worth adding that the MD simulations results have shown that nearly 57% of the total vacancy concentration given in Fig. 5a is likely to be in the form of clusters. However, the number and size of these clusters have not been analyzed.

As the implantations were performed with a consistent ion energy and dose, the significantly higher density of Ga and N atoms displaced in the knock-on collisions induced by a Mg ion compared to that displaced in the collisions induced by a Si ion can be due to the distinguishing features of Mg atoms. The SRIM modelling of the ion/target lattice interaction has shown that there are two stopping mechanisms for an ion incident upon a target lattice, namely electronic and nuclear stopping, and the resultant crystal lattice damage is dependent on the atomic mass and radius^13^. The atomic masses of Si and Mg are 28.086 and 24.305 amu and their covalent atomic radii are 114 and 140 pm, respectively^12^. According to the modelling results the increase of the atomic mass from 24 to 29 amu has negligible effect on the total energy loss to the recoil cascades, however, as the atomic radius increases from 114 to 140 pm, the total energy loss to recoils by the implanted ion rises by more than 40%^13^. Thus, it can be concluded that the substantially higher atomic radius of Mg than that of Si is the main reason that the total vacancy concentration in the Mg-implanted epitaxial GaN is significantly higher compared to that in the Si-implanted material (Fig. 5a).

The predictions of the total vacancy concentrations in the Si- and Mg-implanted epitaxial GaN stated on the grounds of the MD simulation results can be experimentally verified by the comparison with the concentrations of the deep traps identified with the *V*_Ga_ and *V*_N_, found in the samples implanted with Si and Mg ions using the LPITS technique. The results clearly indicate that the concentration of each deep trap in the Mg-implanted sample is higher than that in the Si-implanted one. An important fact worth emphasizing is that there are various differences between the concentrations of the same traps in the both kinds of samples. The smallest difference is for the trap T2 (512 meV), whose concentrations in the Si- and Mg-implanted samples are 7.7 × 10^17^ and 8.7 × 10^17^ cm^−3^, respectively. This trap is attributed to the nitrogen vacancy that in the as-implanted samples acts as a singly ionized donor *V*_N_ ( +) (Table 1). It is easy to notice that this defect is found to be the predominant in the Si-implanted material (Fig. 5b). On the other hand, the largest difference is for the trap T4 (587 meV), whose concentrations in the Si- and Mg-implanted samples are 1.2 × 10^17^ and 7.7 × 10^17^ cm^−3^, respectively. This trap is assigned to the gallium vacancy that in the as-implanted samples plays the role of a doubly ionized acceptor *V*_Ga_ (2−). The small concentration of the trap T4 (587 meV) results from the fact that the vast majority of Si atoms fill the gallium vacancies, forming shallow donors Si_Ga_ ( +). The reaction of interstitial Si atoms with gallium vacancies is energetically favorable because the covalent atomic radius of Si (114 pm) is smaller than that of Ga (123 pm). The covalent atomic radius of Mg (140 pm) is substantially larger than that of Ga and replacing the Ga atoms by this dopant, resulting in the formation of Mg_Ga_ (−) acceptors, requires an additional energy that is provided during post-implantation annealing^2,3^. The difference between the concentrations of the nitrogen split interstitials N_*i*_–N_*i*_ ( +), observed in the Si- and Mg-implanted samples as the trap T3 (531 meV), is not large. The concentrations of this trap in the both kinds of samples are 5.4 × 10^17^ and 8.3 × 10^17^ cm^−3^, respectively. However, in the case of gallium interstitials Ga_*i*_ (2 +), detected in the implanted samples as the trap T6 (736 eV), the concentrations in the Si- and Mg-implanted samples differ by a factor of 3.5. According to the data given in Fig. 5b, these concentrations are 3.4 × 10^17^ and 1.2 × 10^18^ cm^−3^, respectively. The traps properties, listed in Table 1, and concentrations shown in Fig. 5b, were determined in the tail regions of the Si- and Mg-implanted samples. According to the SIMS (Fig. 1) and MD simulations results (Fig. 5a), these regions are located at the depths of 500 and 600 nm, respectively, where the total vacancy concentrations are of the order of 10^18^ cm^−3^. It is worth adding that UV radiation with the wavelength of 375 nm used during the LPITS measurements easily reached these depths in order to generate the excess charge carriers and change the deep traps occupation. The measurements were performed in in the temperature range of 300–520 K and the GaN absorption coefficient values for this wavelength increase with temperature from ~ 1.1 × 10^4^ to ~ 1.55 × 10^4^ cm^−1^, corresponding to the radiation penetration depth changes from ~ 900 to 645 nm^14,15^. The total vacancy concentrations determined experimentally in the tail regions of the Si- and Mg-implanted epitaxial GaN samples are given by the sums of the concentration of trap T2, attributed to the *V*_N_ (2 + / +), and the concentration of trap T4, attributed to the *V*_Ga_ (3−/2−). The sums of the T2 and T4 traps concentrations found from the LPITS measurements in the GaN epitaxial samples implanted with Si and Mg ions are 8.9 × 10^17^ and 1.64 × 10^18^ cm^−3^, respectively. These experimentally determined values are in very good agreement with the total vacancy concentrations in the implanted films at the depths of ~ 500 nm and ~ 600 nm (Fig. 5a), respectively, predicted by the MD simulations. These simulations have also shown that the concentrations of N_*i*_–N_*i*_, *V*_N_, *V*_Ga_, and Ga_i_ in the Si- and Mg-implanted films represent the fractions of the total vacancy concentrations which are 0.012, 0.41, 0.59, and 0.56, respectively. Using these data and the defects concentrations determined experimentally by LPITS studies, we have calculated the total vacancy concentrations in the both kinds of the implanted films. The results follow the total vacancy concentrations shown in Fig. 5a and in the case of the Si-implanted material, the match is found in the region between the depths 460–510 nm, within which the total vacancy concentration decreases from 4.50 × 10^19^ to 2.03 × 10^17^ cm^−3^. For the Mg-implanted material, the match is found in the region located deeper, between the depths of 550–600 nm, within which the total vacancy concentration decreases from 6.92 × 10^19^ to 1.64 × 10^18^ cm^−3^.

## Conclusions

The unique investigations aimed at revealing the properties and concentrations of deep-level defects arising in UID epitaxial GaN during implantations of Si and Mg ions have been performed by using the state-of-the-art LPITS technique and MD simulations. The activation energies, charge carriers capture cross-sections, and concentrations of implantation-induced defects were determined in the tail regions of the as-implanted layers. In the both kinds of implanted layers, the same six deep traps with the activation energies of 396, 512, 531, 587, 635, and 736 meV, respectively, were detected. By comparing the traps activation energies with the reported energy levels for the charge state changes of native defects in GaN, calculated using the HSE hybrid functional, the implantation-induced traps were identified with the interstitials and vacancies at various charge states, namely N_*i*_–N_*i*_ (−/0), *V*_N_ (2 + / +), N_*i*_–N_*i*_ (2 + / +), *V*_Ga_ (3−/2−), *V*_N_ (3 + /2 +), and Ga_*i*_ (+ /2 +), respectively. In the Si-implanted material, the concentrations of these defects were found to be 1.5 × 10^17^, 7.7 × 10^17^, 5.4 × 10^17^, 1.2 × 10^17^, 2.3 × 10^17^, 3.4 × 10^17^ cm^−3^, respectively. In the Mg-implanted material, the defects concentrations were found to be higher by factors of 1.53, 1.13, 1.54, 6.42, 2.70, and 3.53, respectively. The biggest difference is between the *V*_Ga_ concentrations because in the Si-implanted material a large majority of *V*_Ga_ can be filled with Si atoms, forming the shallow donors Si_Ga_.

The GaN lattice defects, identified on the grounds of the LPITS results, are consistent with those predicted by the MD simulations of the lattice damage produced by the ions implantations. According to the simulated results, the lattice damage includes both the areas with the presence of isolated point defects, where the crystalline order is maintained, as well as the highly disordered regions, made up of clusters formed due to the aggregation of various kinds of vacancies and interstitials. The main implantation-induced point defects are vacancies in the gallium and nitrogen sublattices (*V*_Ga_ and *V*_N_) as well as gallium and nitrogen interstitials (Ga_*i*_ and N_*i*_). Among other point defects there are gallium and nitrogen antisites (Ga_N_ and N_Ga_), N_*i*_–N_*i*_ pairs, Ga_*i*_–Ga_*i*_ pairs, and Ga_*i*_–N_*i*_ pairs. The clusters are likely to be created in the regions located not deeper than at ~ 400 nm, where the total vacancy concentrations, being the sums of the *V*_Ga_ and *V*_N_ concentrations, in the Si and Mg-implanted layers exceed the values of ~ 1.0 × 10^21^ and ~ 3.0 × 10^21^ cm^−3^, respectively.

In the Mg-implanted material, the total vacancy concentration is found to be significantly higher than that in the Si-implanted one. It is argued that much higher numbers of Ga and N atoms displaced in the knock-on collisions induced by a Mg ion than by a Si one is due to the substantially larger covalent atomic radius of Mg (140 pm) compared to that of Si (114 pm). Because of the much larger atomic size of Mg, the total energy loss to the recoil cascades can be significantly more efficient.

The MD simulations have shown that the concentrations of N_*i*_–N_*i*_, *V*_N_, *V*_Ga_, and Ga_i_ in the Si- and Mg-implanted layers represent the fractions of the total vacancy concentrations, which for these defects are 0.012, 0.41, 0.59, and 0.56. Under these conditions, the tail region in the Si-implanted material where the defects concentrations are determined experimentally by the LPITS studies is located between the depths 460–510 nm and the total vacancy concentration in this region decreases from 4.50 × 10^19^ to 2.03 × 10^17^ cm^−3^. In the Mg-implanted material the tail region is found to be located deeper, between the depths of 550–600 nm, and the total vacancy concentration within it decreases from 6.92 × 10^19^ to 1.64 × 10^18^ cm^−3^.

## Methods

The 1.5-μm thick UID GaN epitaxial film subjected to implantations of Si and Mg ions was grown by metal–organic chemical vapor deposition (MOCVD) on the (0001) plane of a 2-inch GaN/sapphire template wafer. The epitaxial growth was carried out in an Aixtron close-coupled showerhead MOVPE reactor (CCS 3 × 2&#8221) at the atmospheric pressure and temperature of 1080 °C. Trimethylgallium (TMGa) was used as a Ga precursor, ammonia as a nitrogen source and H_2_ as a carrier gas. According to the Hall-measurements results, the film was *n*-type with a residual net donor concentration of ~ 1 × 10^16^ cm^−3^. From the central part of the wafer with the epitaxial film, several chips of 1 × 1 cm^2^ were cut out and used for implantations of Si^+^ and Mg^+^ ions. The ions were implanted at room temperature with an incident angle 7° off the surface normal. The Si-ion implantation was carried out at in two steps: first at 200 keV with a dose of 5 × 10^13^ cm^−2^ and next at 340 keV with a dose of 1.5 × 10^14^ cm^−2^. The Mg-ion implantation was performed in three steps: at 150 keV and 5 × 10^13^ cm^−2^, 210 keV and 1 × 10^13^ cm^−2^, as well as 270 keV and 1.9 × 10^14^ cm^−2^. The depth profiles Si and Mg concentrations were determined by secondary ion mass spectrometry (SIMS) measurements which were performed employing a CAMECA SC Ultra instrument under ultra-high vacuum (UHV), usually of 4 × 10^−10^ mbar, and using the Cs^+^ primary beam. The sheet resistance of the pristine and implanted samples was measured in darkness at 300 K using a Keithley 2636B System SourceMeter. The TDDC and photocurrent transient measurements were carried out in a temperature range of 300–700 K at a voltage of 20 V using a Keithley 428 fast current amplifier. The MD simulations were performed by means of the Large-scale Atomic/Molecular Massively Parallel Simulator (LAMMPS)^16^.

### Supplementary Information

Supplementary Information.

## Data Availability

The datasets generated during and/or analyzed during the current study are available from the corresponding author on reasonable request.
