# Supplementary material for: Deep-level defects induced by implantations of Si and Mg ions into undoped epitaxial GaN
Source: Sci Rep. 2024 Jun 20;14:14272. doi: 10.1038/s41598-024-65142-w (PMC11190271; doi:10.1038/s41598-024-65142-w)
Supplement: Supplementary file 1 — Supplementary Information. [file 41598_2024_65142_MOESM1_ESM.pdf]

Deep-level defects induced by implantations of Si and Mg ions into undoped epitaxial GaN

Paweł Kamiński[1]*, Andrzej Turos[1,2], Roman Kozłowski[1], Kamila Stefańska-Skrobas[2], Jarosław Żelazko[1] and Ewa Grzanka[3]

[1]Łukasiewicz Research Network – Institute of Microelectronics and Photonics, Aleja Lotników 32/46, 02-668 Warszawa, Poland
[2]National Centre for Nuclear Research, ul. Andrzeja Sołtana 7, 05-400 Otwock, Poland
[3]Institute of High Pressure Physics of the Polish Academy of Sciences, ul. Sokołowska 29/37, 01-142 Warsaw, Poland

*pawel.kaminski@imif.lukasiewicz.gov.pl

Preparation of samples

The samples preparation process was fairly complex and involved the following technological operations: growth of the UID GaN epitaxial film, cutting the 2-inch wafer with the film into chips of 1x1 cm^2 in size, implantations of Si and Mg ions into the epitaxial film on the chips, evaporation of the arrays of two Al co-planar ohmic contacts through a molybdenum mask on the implanted epitaxial film surface, and finally dicing the chips into single samples, with dimensions of approximately 4 x 7 mm^2, having two Al contacts of 2.5x2.5 mm^2 in size and spaced 0.7 mm apart. The structure of samples used for the characterization of the as-implanted materials by the electrical measurements is shown in Figure S1.

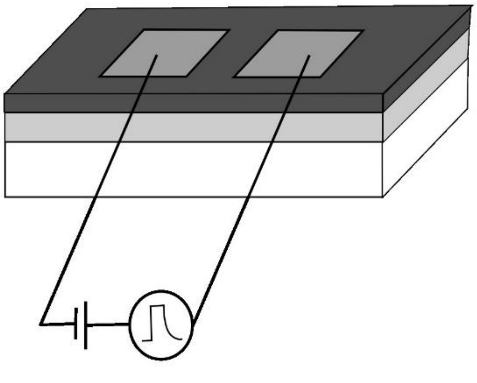

Figure S1. Two-electrode structure of samples prepared for measurements of the electrical properties of the as-implanted films and optically excited photocurrent transients. The size of the 3.31-eV photon laser beam was fitted to the gap between the Al electrodes.

The Al contacts with a thickness of ~300-nm were evaporated in a high vacuum using an electron beam to transform the Al atoms into the gaseous phase and avoid heating the implanted epitaxial film. The Al contacts deposited on the implanted epitaxial film were not subjected to annealing and no thermal processes like bonding were used to attach the electrical leads. All electrical connections to the electrodes were made by mechanical pressure.

At every stage of this process the epitaxial film surface was thoroughly controlled. After the epitaxial growth, which was carried out by MOVPE on a GaN/sapphire epi-ready template, the pristine surface quality was assessed through the observations under an optical microscope and the measurements of the surface roughness using an Atomic Force Microscope (AFM). The former studies indicated that surface smoothness is mirror-like without any faults. According to the AFM measurements, the surface roughness was 0.2 nm. The surface of the implanted epitaxial film on the chips was also mirror-like without any faults. After cutting the chips into the single samples, the surface between the electrodes as well as the sample's edges were thoroughly examined by an optical microscope and only the samples without any faults were taken for the electrical measurements.

Characterization of electrical properties of as-implanted films

Figure S2 shows the I-V characteristics of the UID GaN epitaxial film after implantations of Si and Mg ions. The characteristics were measured at room temperature in darkness for the voltages from -20 to +20 V with an increment of 0.2 V.

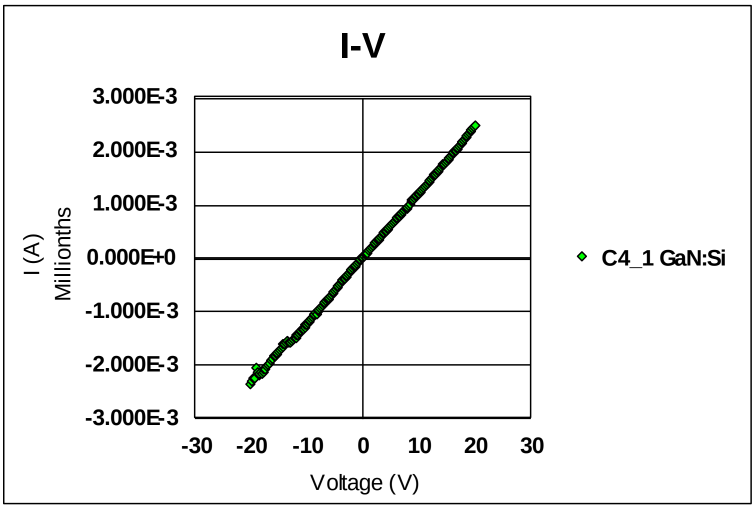

(a)

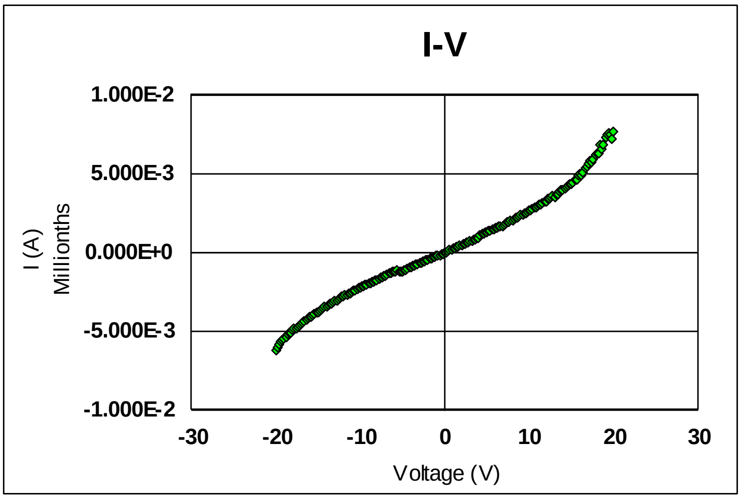

(b)

Figure S2. I-V characteristics measured at room temperature in darkness for Si-implanted (a) and Mg-implanted (b) UID GaN epitaxial film.

It is seen that for the Si-implanted sample, the I-V characteristic is almost perfectly linear in the whole range from -20 to +20 V. The conductance obtained from the slope determined in this range by the linear regression is 1/R = $1.2 \times 10^{-10}\ \Omega^{-1}$ with the perfect $R^2 = 0.999$ and the standard deviation of $2.14 \times 10^{-13}\ \Omega^{-1}$. For the Mg-implanted sample, the I-V characteristic is almost perfectly linear in the range from -10 to + 10 V (Figure S3), however at the voltages near -20 V and +20 V an increase in the slope is clearly visible. The conductance determined by the linear regression from the slope of the characteristic shown in Figure S3 is 1/R = $2.38 \times 10^{-10}\ \Omega^{-1}$ with the very good $R^2 = 0.996$ and the standard deviation of $1.53 \times 10^{-12}\ \Omega^{-1}$. Using these data, the sheet resistance is $1.5 \times 10^{10}\ \Omega$/square.

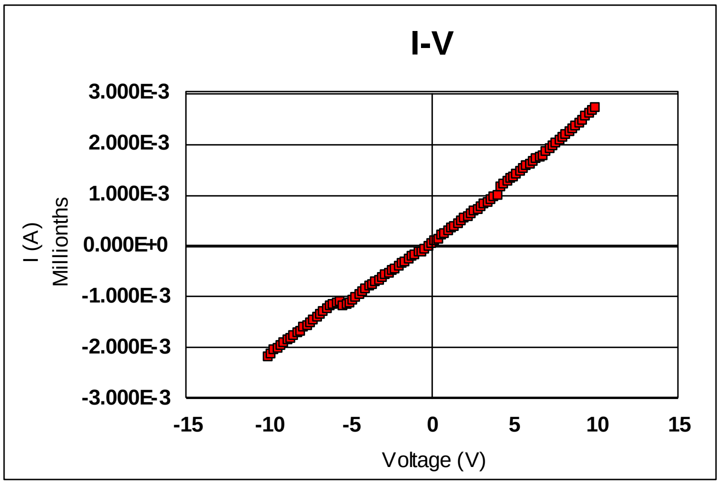

Figure S3. Illustration of the linear part I-V characteristic for the Mg-implanted sample at the voltages from -10 to +10 V.

The SI GaN films after implantations were also characterized by measurements of the temperature dependences of the dark current (TDDC), enabling the activation energy of the films conductivity to be found, and measurements of the mobility-lifetime product ($\mu\tau$) as a function of temperature. For this purpose, a single sample was placed in a thermal chamber equipped with a window enabling the region confined between the two planar contacts to be illuminated with the UV radiation. The TDDC measurements were carried out at temperatures ranging from 300 to 520 K and a voltage of 20 V. The activation energy of dark conductivity (E_{ADC}) was determined from the slope of linear parts of the dark current characteristics, plotted as $T^{3/2}/I$ against 1000/T, where T and I denote the absolute temperature and electrical current, respectively. The temperature dependence of the $\mu\tau$ product was determined in the range of 300 – 520 K by the transient photocurrent method (TPM) [1]. It is based on the assumption that the $\mu\tau$ product at a given temperature is proportional to the height of the photocurrent pulse at the end of the optical excitation pulse. The $\mu\tau$ product values were obtained from the formula [1]

$$\mu\tau = I_{ph}/[\eta(1-R)qlEF], \tag{1}$$

in which I_{ph} is the height of the photocurrent pulse generated by the photon flux F, η is the quantum efficiency of the electron-hole pairs generation, R is the reflection coefficient, q is the elementary charge, l is the contacts width, and E is the applied electric field dependent on the voltage applied between the two contacts on a sample. The photon flux, controlled by dedicated optical filters, was $7.9 \times 10^{16}\ cm^{-2}s^{-1}$ and the electric field was 286 V/cm. The values of η and R were assumed to be 1 and 30 %, respectively. The excitation pulse width and the repetition period were 50 ms and 500 ms, respectively. The excess charge carriers were generated by illuminating the material in the gap between the two contacts with the UV radiation pulses with the wavelength of 375 nm (photon energy 3.31 eV) emitted by a semiconductor laser.

Figure S4(a) shows the comparison of the TDDC measurements results and Fig. S4(b) shows the $\mu\tau$ product temperature dependences determined for the Si- and Mg-implanted samples. The activation energy of conductivity for the former is 480 meV and for the latter it is 745 meV. This energy corresponds to the Fermi level position in the bandgap extrapolated to 0 K [2]. In other words it can be interpreted as the Fermi level position resulting from the ratio of the total (shallow and deep) donor concentration (N_D) to the total (shallow and deep) acceptor concentration (N_A). It is worth adding that for the implanted samples, the N_D is likely to be higher than N_A because to the N_D contribute also the residual shallow donors Si_{Ga} present in in the starting material. Therefore it can be assumed that the activation energies of 480 and 745 meV represent the Fermi level positions in the upper half of the bandgap and the energy values are with respect to the conduction band minimum.

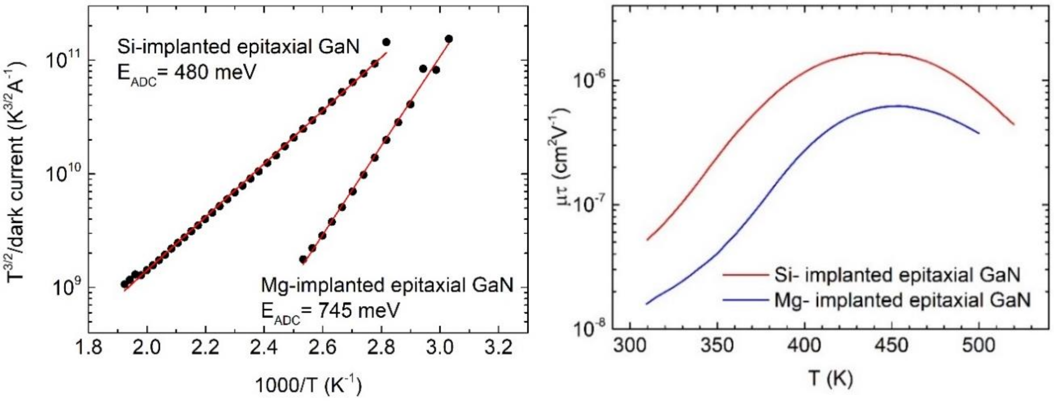

(a) **(b)**

Figure S4. (**a**)Temperature dependences of dark current for samples of SI films made in UID epitaxial GaN by implantations of Si and Mg ions. The activation energies of the films conductivity of 480 meV and 745 meV, respectively, are obtained from the slope of the straight lines fitted by linear regression to the experimental data. (**b**) Temperature dependences of the mobility-lifetime product determined for SI films produced in UID epitaxial GaN by implantations of Si and Mg ions.

In the case of Si-implanted film, the position of the Fermi level is significantly closer to the conduction band minimum compared to that in the Mg-implanted material and this fact indicates that the total donor concentration in the former is much higher than that in the latter. In other words, the donors in the Si-implanted material are substantially less compensated than in the Mg-implanted one. This fact can be well illustrated by the electron concentration values corresponding to the Fermi level positions at 300 K in the both materials. These values are 1.9×10^{10} and 6.9×10^{5} cm^{-3}, respectively, and confirm a much lower compensation level for the Si-implanted film compared to that for the Mg-implanted one. This phenomenon can be accounted for by assuming that during the Si implantation a part of the Si atoms interact with Ga vacancies and the shallow Si_{Ga} donors are formed. This interaction is plausible, since the Si atomic radius (114 pm) is lower than the Ga atomic radius (123 pm) and the energy necessary for substituting the Ga atom by the Si atom in the GaN lattice is very small [3]. The Mg atomic radius (140 pm) is much larger than that of Si and the formation of the Mg_{Ga} acceptors during the implantation is very unlikely. Thus, the charge compensation in the Mg-implanted material is mainly due to the presence of the implantation-induced native defects being deep donors or acceptors [4,5].

The $\mu\tau$ changes induced by increasing the temperature in the range of 300 – 520 K mainly reflect the changes of the excess charge carriers lifetime. The results presented in Fig. 3 indicate that for both the Si- and Mg-implanted samples the $\mu\tau$ product up to 450 K goes up by more than one order of magnitude. The temperature dependence of the charge carriers lifetime can be analyzed using the Shockley-Read-Hall (SRH) recombination model, describing the recombination process of electron-hole pairs with the involvement of deep-level defects [6]. The deep levels can effectively increase the rate of a two-step recombination process where first conduction electrons are captured by the defect

level and then this level captures holes from the valence band. In this way the electron-hole pairs are annihilated through the charge carriers transitions from the valence and conduction bands to the defect level. With increasing temperature, the thermal emission rate of charge carriers from defects levels becomes significant and the two-step recombination process is more and more desynchronized. In other words, the captured electron can be released again to the conduction band before a hole is captured by the defect level. Since the thermal emission rate of charge carriers exponentially increases with rising temperature [6], the desynchronization of the capture end emission processes can explain the strong increase in the lifetime seen in Fig. S4(b). According to the SRH model, the charge carriers lifetime is inversely proportional to the concentration of defect centers taking part in the recombination process [6]. This means that in the Mg-implanted epitaxial GaN the concentration of deep-level defects affecting the lifetime can be approximately two times higher than in the Si-implanted material.

Deriving the traps properties and concentrations from the photocurrent relaxation waveforms

The LPITS measurements involved digital recording of the photocurrent transients generated at temperatures 300 – 520 K with an increment of 5 K by optical pulses with a photon energy of 3.31 eV emitted by the same semiconductor laser used for determining the $\mu\tau$ values. To improve the signal to noise ratio, the digital data were averaged by taking 500 transients. The photon flux was 7.9 × 10^{17} $cm^{-2}s^{-1}$ and the voltage applied between the two coplanar contacts was 20 V. For further processing, each photocurrent transient was normalized with respect to the photocurrent pulse height at the end of the illumination pulse. In order to get the temperature dependences of the thermal emission rate of charge carriers for detected defect levels, the analysis of the of photocurrent relaxation waveforms was performed under the assumption that each of these waveforms observed at a given temperature T is the sum of several exponential components produced by the thermal emission of the excess charge carriers from various energy levels of defect centers [7,8]. It is worth adding that the relaxation waveform induced by the thermal emission of charge carriers trapped under illumination represents only a slower part of the photocurrent decay occurring after switching off the excitation pulse. The time constants of the exponential components whose sum is observed as a photocurrent relaxation waveform are longer than ~1 µs. The faster part of the photocurrent decay of less than 1000 ns duration is mainly due to the excess charge carriers recombination [7,9]. At a temperature T, an exponential component of the photocurrent relaxation waveform can be written as [7]

$$I(t) = I(0)\exp(-e_T t), \tag{2}$$

where e_T is the charge carriers thermal emission rate characteristic of the defect center that captured these carriers during a sample illumination and $I(0)$ is the amplitude of the exponential signal when the optical excitation pulse is terminated. The e_T is equal to the reciprocal of the relaxation signal time constant and the $I(0)$ can be expressed in the form

$$I(0) = qn_T(0)e_T\mu\tau EC, \tag{3}$$

where $n_T(0)$ is the concentration of electrons or holes trapped by the center when the optical excitation pulse is switched off and C is the geometrical parameter equal to the area of the cross-section of a sample region through which the excess charge carriers emitted from the defect center flow to the electrodes on the sample surface. This parameter is dependent on temperature according to the formula [7]

$$C(T) = (1/\alpha + L_D)l, \tag{4}$$

where α is the temperature dependent absorption coefficient [10,11], l is the electrodes width, and L_D is the temperature dependent charge carriers diffusion length given by $L_D = [(kT/q)\mu\tau]^{1/2}$. The temperature dependence of the thermal emission rate of electron or holes is given by the Arrhenius formula [7]

$$e_T(T) = AT^2\exp(-E_a/kT), \quad (5)$$

where E_a is the activation energy, k is the Boltzmann constant, $A = \gamma\sigma_a$ is the pre-exponential factor, equal to the product of the material constant γ, dependent on the effective mass, and the apparent capture cross-section for electrons or holes σ_a. Thus, each defect center is characterized by the activation energy E_a of the electron or hole thermal emission and pre-exponential factor A. For GaN with wurtzite structure, the values of γ_n and γ_p, necessary to get the apparent capture cross-sections for electrons or holes, are 6.48 × 10^{20} and 6.33 × 10^{21} $K^{-2}cm^{-2}s^{-1}$, respectively.

The concentration (N_T) of a defect center is determined from the amplitude of the exponential component of the photocurrent relaxation waveform observed at a given temperature T. According to the equation (3) the amplitude $I(0)$ is proportional to the concentration $n_T(0)$ of electrons or holes trapped by the center when the optical excitation pulse is switched off. To find the experimental values of $I(0)$ for the exponential signals revealed in the one-dimensional Laplace spectrum resulting from the analysis of the relaxation waveform measured in temperature T, this waveform is fitted by the sum of exponential functions in the form of equation (2) whose number and emission rate values are indicated by the Laplace spectrum. First the $n_T(0)$ is calculated at the temperature T using the formula

$$n_T(0) = I(0)/(qe_TEC\mu\tau). \quad (6)$$

Then the $n_T(0)$ values are determined for several temperatures and the dependence of $n_T(0)$ as a function of e_T is plotted. The trap concentration N_T is determined by the extrapolation of the $n_T(0)$ = f(e_T) plot to the value of $n_T(0)$ for $e_T \rightarrow 0$, according to the formula [7]

$$n_T(0) = N_T/(1+ e_T/G\tau c_T), \quad (7)$$

where G, τ, and c_T are the excess charge carriers generation rate, lifetime, and capture coefficient, respectively.

To reveal the number of exponential components described with equation (2) in the photocurrent relaxation waveform measured at a given temperature T_j and to determine the thermal emission rate values for defect centers from which the charge carriers are released, the advanced numerical procedure based on the inverse Laplace transformation algorithm (ILT) implemented in the CONTIN code is used [12]. As a result, the one-dimensional (1D) Laplace spectrum $S_{Lj}(e_T)$ with sharp peaks seen at various values of the thermal emission rate characteristic for the defect centers detected in this temperature is obtained. In order to get the temperature dependences of the emission rate for all defect centers contributing to the thermal emission of charge carriers all the 1D Laplace spectra resulted from the analysis of the photocurrent relaxation waveforms recorded at all temperatures T_j (j = 1, 2, 3 …..N) are assembled. In this way the two-dimensional (2D) Laplace spectrum is created, which in 3D space has the form of the sharp folds whose ridgelines depict the temperature dependences of the emission rate for the defect centers that trapped the charge carriers during the sample illumination [9]. In practice, the folds are projected on the plane given by the temperature - emission rate (T, e_T) axes and projections of their ridgelines are used to determine the temperature dependences of emission rate for detected traps. These dependences are used for drawing the Arrhenius plots and determining the traps activation energies E_a and values of the pre-exponential factor in the Arrhenius equation. The folds projected on the (T, e_T) plane are called the Laplace spectral fringes.

Images of the Laplace spectral fringes for defect centers detected by the two-dimensional analysis of the photocurrent relaxation waveforms recorded in the temperature range of 320 – 520 K for samples implanted with Si and Mg ions are shown in Figure S5. The images in Figs. S5(a) and S5(b) are very similar, which indicates that in the both samples the same deep-level defects are present. For the particular defects, labelled as T1, T2, T3, T4, T5, and T6, the Laplace spectral fringes occur in various temperature ranges and the various values of the charge carriers emission rate are observed. This fact is due to the various properties of defect centers. The effect of recombination process should

also be taken into account, since the thermal emission is represented only by a part of the relaxation waveform. According to the images shown in Fig. S5, the thermal emission for theT1 trap is observed at temperatures 320 – 375 K for emission rates 3162 – 39811 s^{-1}. For the traps T2, T3, T4, T5, and T6 the temperature ranges are 320 – 390 K, 320 – 430 K, 380 – 490 K, 420 – 500 K, and 450 – 500 K, respectively, and the emission rate values are 316 – 31623 s^{-1}, 100 – 31623 s^{-1}, 501 – 31623 s^{-1}, 1000– 19953 s^{-1}, and 1000 – 10000 s^{-1}, respectively. It is worth noting that using the CONTIN program

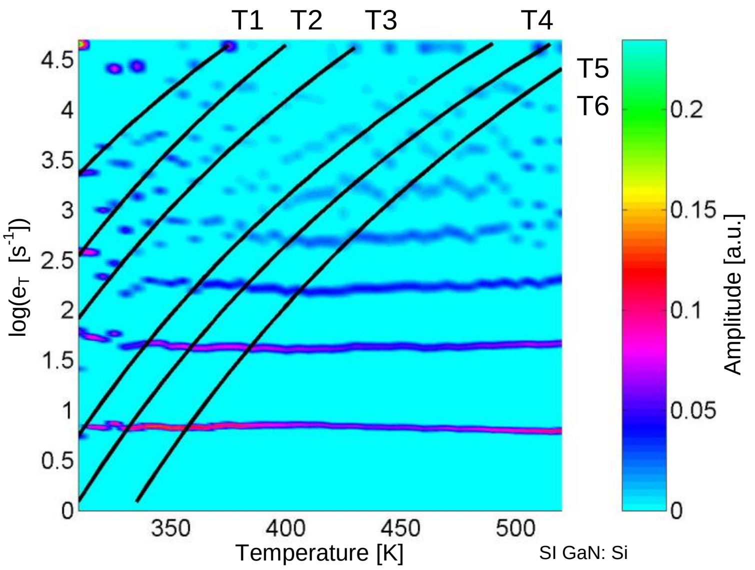

(a)

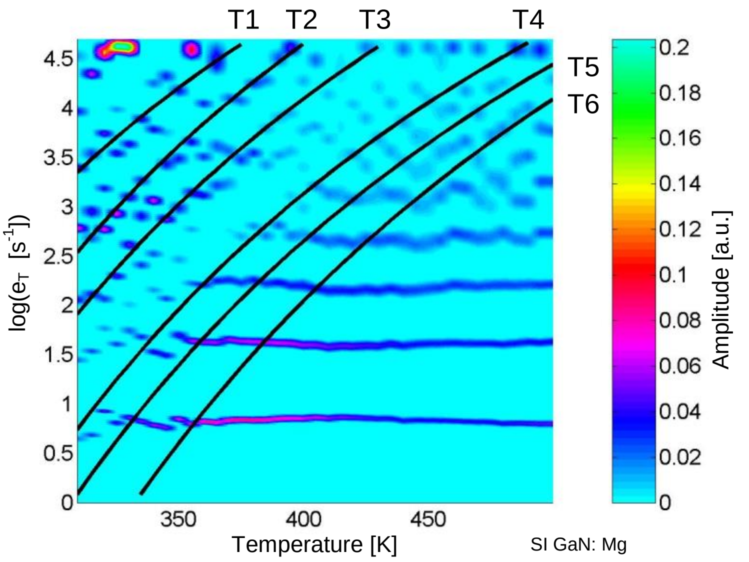

(b)

Figure S5. Images of the Laplace spectral fringes for defect centers, labeled as T1, T2, T3, T4, T5, and T6, detected in in UID GaN epitaxial film implanted with Si **(a)** and Mg ions **(b)**. The solid lines, obtained by the fringes approximation with the Arrhenius equation, illustrate the temperature dependences of the charge carriers emission rate.

2D analysis of the photocurrent relaxation waveforms can give the results not only associated with the exponential components related to the thermal emission, but also it can reveal the components induced by the surface recombination whose time constants are not temperature dependent. In this

case the results are independent of temperature and indicate a single emission rate value. The presence of these components is clearly reflected in the images shown in Fig. S5. The solid lines seen in Fig. S5 are the results of the best fit of the Arrhenius formula to the data of the Laplace spectral fringe for each trap.

Traps properties and concentrations

The Arrhenius plots, characterizing the temperature dependences of the charge carriers thermal emission rate for the six deep traps T1 – T6 revealed in the Si- and Mg-implanted UID epitaxial films, are shown in Fig. S6.

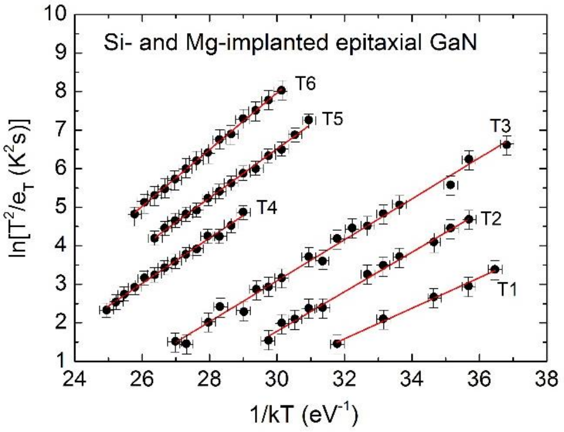

Figure S6. Arrhenius plots illustrating the dependences of the reciprocal of the emission rate of charge carriers as a function of the reciprocal of the thermal energy for six deep traps, labelled as T1, T2, T3, T4, T5, and T6, revealed by the LPITS measurements in the semi-insulting Si- and Mg-implanted films in UID epitaxial GaN. The straight lines are fitted by linear regression to the experimental data.

The plots were created using the data of the Laplace spectral fringes shown in Fig. S5. They depict the reciprocals of the thermal emission rate as a function of the reciprocal of thermal energy. For the traps located deeper in the bandgap, a higher thermal energy must be provided to induce the thermal emission of electrons or holes to the conduction or valence band, respectively. Each Arrhenius plot is the trap signature and its slope allows determining the trap activation energy and the intercept gives the pre-exponential factor in the Arrhenius equation. It worth emphasizing that the Arrhenius plots for the T2 and T3 traps as well as those for the T4 and T5 traps are separated owing to a much higher resolution of the LPITS technique compared to the conventional PITS or DLTS techniques in which the correlation procedure based on the box-car analysis of the relaxation waveforms is used [8,13,14]. The deep traps parameters derived from the Arrhenius plots presented in Fig. S6, as well as the traps concentrations determined for the samples of Si- and Mg-implanted UID epitaxial GaN from the amplitudes of the exponential components found in the photocurrent relaxation waveforms are listed in Table 1.

Trap label	E_a [meV]	A [$s^{-1}K^{-2}$]	Trap concentration [cm^{-3}]	
			Si-implanted sample	Mg-implanted sample
T1	396 ± 10	6.6×10^4	1.5×10^{17}	2.3×10^{17}
T2	512 ± 8	7.9×10^5	7.7×10^{17}	8.7×10^{17}
T3	531 ± 8	3.8×10^5	5.4×10^{17}	8.3×10^{17}
T4	587 ± 10	2.1×10^5	1.2×10^{17}	7.7×10^{17}
T5	635 ± 10	2.8×10^5	2.3×10^{17}	6.2×10^{17}
T6	736 ± 8	1.3×10^6	3.4×10^{17}	1.2×10^{18}

Table S1. Summary of the activation energies (E_a) , pre-exponential factors (A) in the Arrhenius equation and concentrations of deep traps detected by LPITS in semi-insulating Si- and Mg-implanted films made in UID epitaxial GaN.

In view of the results of simulations performed using MD methods the main defects produced during ion implantations are Frenkel pairs [15,16]. Therefore, the traps whose parameters and concentrations are listed in Table 1 are expected to be related to the native defects formed in the Ga and N sublattice due to collisions of the implanted ions with the Ga and N atoms located in the substitutional sites. Thus, the traps detected by the LPITS are likely to be related to gallium vacancies (V_{Ga}), gallium interstitials (Ga_i), nitrogen vacancies (V_N), and nitrogen interstitials (N_i).To assign each trap to the particular lattice defect in wurtzite GaN, the trap activation energies were compared with the transition energies, established by hybrid density-functional calculations, corresponding to the charge state changes of the native defects in the material [17]. In this way the T1 trap activation energy of 396 meV can be tentatively identified with the acceptor level of the split interstitial (N_i - N_i), located in the bandgap at E_c – 352 meV and corresponding to the (-/0) charge state change. The split interstitial is formed by two nitrogen atoms sharing a lattice site with the bond length between them of 0.11 – 0.14 nm [17] and it exhibits the lowest formation energy in *n*-type GaN [17,18]. It should be added that comparing the two physical quantities is a complex matter. The reported position of the N_i - N_i (0/-) transition level shows that it is located at 3.02 eV above the valence band maximum (VBM) and the transition is related to the capture of an electron from the valence band [17]. On the other hand, the activation energy obtained from the LPITS measurements is assumed to be related to the thermal emission of an electron from the occupied level of the N_i - N_i acceptor to the conduction band and it is measured with respect of the conduction band minimum (CBM). Thus, in order to find the reference value of energy for the N_i - N_i (0/-) transition it is necessary to know the average value of the bandgap width in the temperature range in which the electron thermal emission is observed. According to the results shown in Fig. S5, the thermal emission of charge carriers from the T1 trap occurs in the temperature range of 320 – 375 K and the average bandgap width value in this range is 3.372 eV [10], which after subtracting 3.02 eV gives the value of 352 meV below the CBM. Moreover, when the charge carriers capture preceding the thermal emission occurs by a multi-phonon process, the activation energy determined from the Arrhenius plot represents the sum of the defect ionization energy and the activation energy for thermal capture an electron from the conduction band, or a hole capture from the valence band [19]. In other words, the activation energy derived from LPITS or DLTS studies can be by 30 – 60 meV higher than the calculated energy required for the defect charge state change [19].

The T2 trap activation energy of 512 meV very well matches the donor level of nitrogen vacancy (V_N) located at E_v + 470 meV. The level is related to the hole emission resulting in the (2+/+) charge state change. The calculated (2+/+) transition energy in this case is given with respect of the VBM [17] and the comparison can be made directly. For the T2 trap, the electron thermal emission is observed in the temperature range of 320 – 390 K (Fig. S5). Before the sample illumination, the V_N in these temperatures is singly positively ionized and when it captures an excess hole from the valence band under the illumination it becomes doubly positively ionized. When the optical excitation is terminated, the hole is thermally emitted to the valence band and the defect returns to its starting charge state. In turn the T3 trap activation energy of 531 meV can be attributed to the N_i – N_i donor level located at E_v + 510 meV related to the (2+/+) transition involving a hole thermal emission [17]. This emission is observed in the temperature range of 320 – 430 K after the excess hole capture from the valence band during the sample illumination. The T4 trap with the activation energy of 587 meV is likely to be the electron trap that can be assigned to the (3-/2-) transition level of the V_{Ga} acceptor [17]. Since the reported energy level is given at 2.8 eV above the VBM for the V_{Ga} (2-/3-) transition related to the capture of an electron from the valence band [17], it was necessary, similarly as in the case of the T1 trap, to find the adequate energy for the electron emission to the conduction band. Taking into account that the thermal emission of excess charge carriers from the T4 trap is observed in the temperature range of 380 – 490 K (Fig. S5) and assuming that the average bandgap width in this range is 3.326 eV [10], the energy necessary for the V_{Ga} (3-/2-) transition and release an electron to the conduction band is 526 meV. The T5 trap activation energy of 635 meV can be identified with the donor level of V_N located at E_v + 610 eV and related to the (3+/2+) charge state transition involving a hole emission to the valence band [17]. Finally, the T6 trap activation energy of 736 meV is attributed to the donor level of Ga_i located at E_c – 696 meV and related to the (+/2+) charge state

change involving an electron emission. This identification is based on the result of calculations showing that the level for Ga_i (2+/+) transition is located at 2.64 eV above the VBM [17]. Taking into account that the thermal emission of excess charge carriers from the T6 trap occurs in the temperature range of 450 – 500 K and the average value of the bandgap width in this range is 3.336 eV, the position of the Ga_i (+/2+) level related to the electron emission is at 696 meV below the VBM.

Methodology of molecular dynamics simulations

To confirm the atomic configurations and concentrations of defects produced in the collision cascades in UID epitaxial GaN under the ion bombardment we performed the MD simulations using a model of the GaN crystal lattice consisting of 163200 atoms which relaxed at room temperature after stopping cascades of collisions and the Frenkel pairs recombination. It was assumed that the cascade starts from the ejection of a single Ga atom from the lattice site and the kinetic energy values as well as the velocity direction can be randomly selected. The energy of a knocked-on Ga atom ranged up to 400 keV and the deflection from the <0001> direction varied in the range of 0 – 90 degrees. The azimuth angle was in the range from 0 to 60 degrees. The MD simulations in this study were performed using the Large-scale Atomic/Molecular Massively Parallel Simulator (LAMMPS) cod [20]. To calculate the bond order potentials for the Ga_i-Ga_i, N_i-N_i, and Ga_i-N_i interactions, the parameter sets reported by Nord *et al*. [21] were used. Geometric models of defects atomic configurations resulting from the implantation-induced GaN lattice damage were determined and visualized by the Voronoi cell finite element method [22]. This method is based on computational geometry where the spatial structure of defects is approximated by balls representing individual atoms. The calculations were performed in three stages. First the models of three types of primary defects, namely interstitial atoms, vacancies, and anti-sites arising in both the Ga and N sublattice were created. Next, the interaction between the interstitial atoms has been taken into account and the models for the four groups of defects were calculated. These are isolated interstitial atoms, in the vicinity of which there are no neighbors at a distance less than 0.319 nm, as well as pairs of Ga_i-Ga_i, Ga_i-N_i, and N_i-N_i atoms, with the bond lengths of 0.197, 0.193, and 0.115 nm, respectively, creating dumbbells. Finally, the defects clusters consisting of more than one displaced Ga or N atoms were modelled.

Impact of the implantation-induced defects on the dopants depth profiles

Controlling the dopants concentration profiles is an important issue in fabrication of GaN devices. Usually the dopants concentration profiles are predicted by using SRIM simulations based on the theoretical assumptions. We have performed the SRIM simulations which showed that at the conditions selected for the implantations of Si and Mg ions, the n^+ and p^+ films with uniform Si and Mg concentrations of 7.1×10^{17} and 7.5×10^{17} cm^{-3}, respectively, located at the depth of 200 – 300 nm from the surface of UID epitaxial film, could be produced. According to the SIMS results, the targeted Si concentration was achieved only in the Si implanted material, in which the region with the uniform dopant concentration extended from the depth of ~150 nm to 350 nm. In the Mg-implanted epitaxial film, the measured uniform Mg concentration was ~ 4×10^{17} cm^{-3}, being nearly two times lower than that predicted by the SRIM simulations. Moreover, the region with the nearly constant Mg concentration was much narrower, being in the range of depths 150 – 250 nm. Thus, the above-mentioned data clearly show that in contrast to the implantation of Si ions, in the case of implantation of Mg ions, the SRIM simulations results significantly differ from that obtained experimentally. In other words, the theoretical calculations do not take into account the fact that the concentration of the lattice defects produced under the Mg ions implantation is significantly higher than that of the defects formed under the implantation of Si ions. Therefore, the lower Mg concentration in the as-implanted material results from a lower efficiency of the Mg ions incorporation compared to the efficiency of the Si ions. This is because a significantly larger part of the Mg ion energy is used for the generation of defects and due to a much higher concentration of the implantation-induced defects that prevent from the Mg ion movement deep into the material. Since the implanted ions are incorporated interstitially, the concentration of native interstitials, such as N_i and Ga_i, which block the movement of Mg ions, is of great importance. In particular, the large atomic radius (123 pm) of Ga

makes that Ga_i can act as an efficient blocker. According to the LPITS results, the Ga_i concentrations in the Si- and Mg-implanted layers are 3.4x10^{17} and 1.2x10^{18} cm^{-3}, respectively. The dopant depth profiles change after annealing which is performed to produce shallow donors Si_{Ga} and shallow acceptors Mg_{Ga}. During the annealing process, Mg atoms diffuse in damaged regions introduced by ion implantation, and they interact with various defects such as vacancies and interstitials. Therefore, knowledge on interactions between Mg and point defects is central to controlling not only the activation of Mg, but also the dopant depth profile. The LPITS results indicate that the Mg depth profile can be changed during annealing due to the formation of Mg_{Ga} - V_N complexes. The V_N concentration in the Mg-implanted layer of UID epitaxial GaN is 6.2x10^{17} cm^{-3} and this value is sufficiently high to form these complexes.

References

[1] Schwartz, W.R. & Haegel, N.M. Direct determination of the mobility-lifetime (ls) product from the transient response of extrinsic Ge:Ga photoconductors. *Infrared Physics & Technology* **45** 125–129 (2004). doi:10.1016/j.infrared.2003.07.004

[2] Darling, RB. Electrostatic and current transport properties of n^+/semi- insulating GaAs junctions. *J. Appl. Phys.* **74**, 4571–4589 (1993). doi.org/10.1063/1.354376

[3] Mantina, M., Valero, R., Cramer, Ch. J. & Truhlar, D. G. Atomic radii of the elements. *CRC Handbook of Chemistry and Physics*; Editors: Haynes, W. M., Lide, D.R. and Bruno, T. J., CRC Press Taylor & Francis Group 6000 Broken Sound Parkway NW, Suite 30, p. 9-50 (2014).

[4] Roccaforte, F., Giannazzo, F. & Greco, G. Ion Implantation Doping in Silicon Carbide and Gallium Nitride Electronic Devices. *Micro* **2**, 23–53 (2022). doi.org/10.3390/micro2010002

[5] Xiao, H. Y., Gao, F., Zu, X. T. & Weber, W. J. Threshold displacement energy in GaN: *Ab initio* molecular dynamics study. *J. Appl. Phys.* **105**, 123527 (2009). doi.org/10.1063/1.3153277

[6] Zhang, J. *et al*. Effect of point defects trapping characteristics on mobility-lifetime (μτ) product in CdZnTe crystals. *Journal of Crystal Growth* **519**, 41-45 (2019). doi.org/10.1016/j.jcrysgro.2019.04.026

[7] Kamiński, P. *et al*. Investigation of Energy Levels of Small Vacancy Clusters in Proton Irradiated Silicon by Laplace Photoinduced Transient Spectroscopy. *Crystals* **12**, 1703 (2022). doi.org/10.3390/cryst12121703

[8] Kruszewski, P. *et al*. Laplace DLTS studies of the 0.25 eV electron trap properties in n-GaN. *Semicond. Sci. Technol*. **36,** 035014 (2021). doi.org/10.1088/1361-6641/abe31

[9] Kamiński, P., Kozłowski, R., Miczuga, M. *et al*. High-resolution photoinduced transient spectroscopy of defect centers in vanadium-doped semi-insulating SiC. *J Mat. Sci. Materials in Electronics* **19**, 224-228 (2008). 10.1007/s10854-008-9576-6

[10] Teisseyre, H. Perlin, P. Suski, T. *et al*. Temperature dependence of the energy gap in GaN bulk single crystals and epitaxial layer. *J. Appl. Phys.* **76**, 2429-2434 (1994). doi.org/10.1063/1.357592

[11] Muth, J. F. *et al*. Absorption coefficient, energy gap, exciton binding energy, and recombination lifetime of GaN obtained from transmission measurements. *Appl. Phys. Lett.* **71**, 2572-2574 (1997). doi.org/10.1063/1.120191

[12] Provencher, S. W. CONTIN: a general purpose constrained regularization program for inverting noisy linear algebraic and integral equations. *Comp. Phys. Comm*. **27**, 229-242 (1982). doi.org/10.1016/0010-4655(82)90174-6

[13] Chen, X. D. *et al*. Deep level defect in Si-implanted GaN n^+-p junction. *Appl. Phys. Lett*. **82**, 3671-3673 (2003). doi.org/10.1063/1.1578167

[14] Alfieri, G., Sundaramoorthy, V. K. & Micheletto, R. Electrically active point defects in Mg implanted n-type GaN grown by metal-organic chemical vapor deposition. *J. Appl. Phys.* **123**, 205303 (2018). doi.org/10.1063/1.5029254

[15] Kucheyev, S.O., Williams, J.S. & Pearton, S.J. Ion implantation into GaN. *Mat. Sci. Eng.* **33,** 51-107 (2001). doi.org/10.1016/S0927-796X(01)00028-6

[16] Qiu, R. *et al*. Molecular dynamics simulations of displacement cascades in vanadium: Generation and types of dislocation loops. *Nuclear Materials and Energy* **34,** 101394 (2023). doi.org/10.1016/j.nme.2023.101394

[17] Diallo, I. C. & Demchenko, D. O. Native Point Defects in GaN: A Hybrid-Functional Study. *Phys. Rev. Applied* **6**, 064002 (2016). doi.org/10.1103/PhysRevApplied.6.064002

[18] Lyons, J. L. & Van de Walle, Ch. G., Computationally predicted energies and properties of defects in GaN. *npj Computational Materials* **3:12**, (2017). doi:10.1038/s41524-017-0014-2

[19] Mooney P. M. Deep donor levels (*DX* centers) in III-V semiconductors. *J. Appl. Phys*. **67**, R1-R26 (1990). doi.org/10.1063/1.345628

[20] Thompson, A. P., Aktulga, H.M., Berger, R. *et al*. LAMMPS - a flexible simulation tool for particle-based materials
modeling at the atomic, meso, and continuum scales. *Computer Physics Communications* **271**, 108171 (2022). doi.org/10.1016/j.cpc.2021.108171

[21 Nordy, J., Albez, K., Erhartzand, P. & Nordlund, K. Modelling of compound semiconductors: Analytical bond-order potential for gallium, nitrogen and gallium nitride. *J. Phys.: Condens. Matter* **15**, 5649 (2003). doi.org/10.1088/0953-8984/15/32/324

[22] Manaka, M. *et al*. Hybrid Voronoi diagrams, their computation and reduction for applications in computational biochemistry. *Journal of Molecular Graphics and Modelling* **74,** 225-233 (2017). https://doi.org/10.1016/j.jmgm.2017.03.018

Legend

Page 8, **Table S1.** Summary of the activation energies (E_a) , pre-exponential factors (A) in the Arrhenius equation and concentrations of deep traps detected by LPITS in semi-insulating Si- and Mg-implanted films made in UID epitaxial GaN.
Page 1, **Figure S1.** Two-electrode structure of samples prepared for measurements of the electrical properties of the as-implanted films and optically excited photocurrent transients. The size of the 3.31-eV photon laser beam was fitted to the gap between the Al electrodes.
Page 2, **Figure S2.** I-V characteristics measured at room temperature in darkness for Si-implanted (a) and Mg-implanted (b) UID GaN epitaxial film.
Page 3, **Figure S3.** Illustration of the linear part I-V characteristic for the Mg-implanted sample at the voltages from -10 to +10 V.
Page 4, **Figure. S4.** (**a**)Temperature dependences of dark current for samples of SI films made in UID epitaxial GaN by implantations of Si and Mg ions. The activation energies of the films conductivity of 480 meV and 745 meV, respectively, are obtained from the slope of the straight lines fitted by linear regression to the experimental data. (**b**) Temperature dependences of the mobility-lifetime product determined for SI films produced in UID epitaxial GaN by implantations of Si and Mg ions.
Page 7, **Figure S5.** Images of the Laplace spectral fringes for defect centers, labeled as T1, T2, T3, T4, T5, and T6, detected in in UID GaN epitaxial film implanted with Si **(a)** and Mg ions **(b)**. The solid lines, obtained by the fringes approximation with the Arrhenius equation, illustrate the temperature dependences of the charge carriers emission rate.
Page 8, **Figure S6.** Arrhenius plots illustrating the dependences of the reciprocal of the emission rate of charge carriers as a function of the reciprocal of the thermal energy for six deep traps, labelled as T1, T2, T3, T4, T5, and T6, revealed by the LPITS measurements in the semi-insulting Si- and Mg-implanted films in UID epitaxial GaN. The straight lines are fitted by linear regression to the experimental data.
